# Supplementary material for: A Humanized Yeast Phenomic Model of Deoxycytidine Kinase to Predict Genetic Buffering of Nucleoside Analog Cytotoxicity
Source: Genes (Basel). 2019 Sep 30;10(10):770. doi: 10.3390/genes10100770 (PMC6826991; doi:10.3390/genes10100770)
Supplement: Supplementary file 1 [file genes-10-00770-s001.zip › Additional_Files/Additional_File7_GOTermHeatmaps.zip!/Additional_File7_GOTermHeatmaps/spindle_organization.pdf]

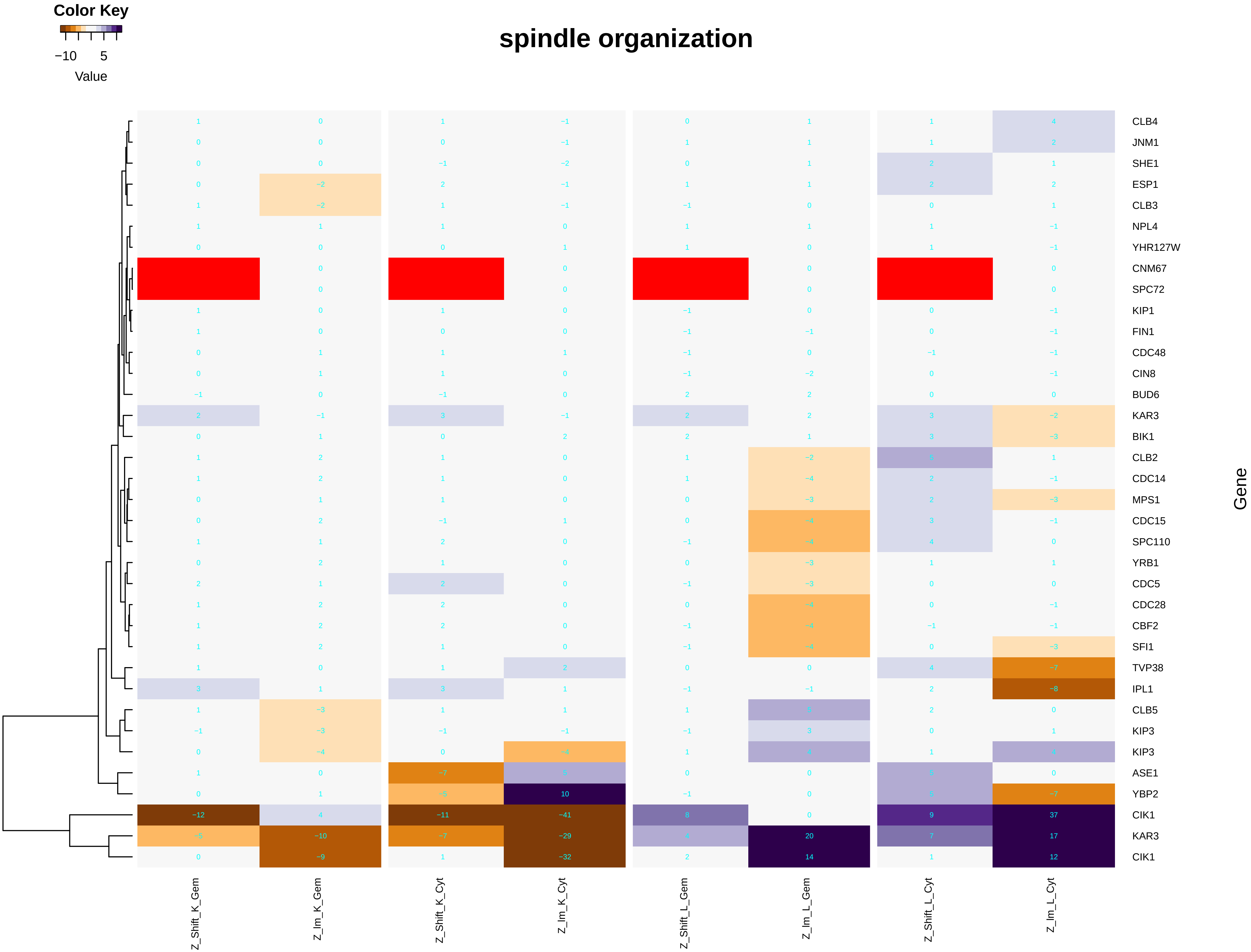

Color Key
−10
5
Value
spindle organization
CLB4
JNM1
SHE1
ESP1
CLB3
NPL4
YHR127W
CNM67
SPC72
KIP1
FIN1
CDC48
CIN8
BUD6
KAR3
BIK1
CLB2
CDC14
MPS1
CDC15
SPC110
YRB1
CDC5
CDC28
CBF2
SFI1
TVP38
IPL1
CLB5
KIP3
KIP3
ASE1
YBP2
CIK1
KAR3
CIK1
Gene
Z_Shift_K_Gem
Z_Im_K_Gem
Z_Shift_K_Cyt
Z_Im_K_Cyt
Z_Shift_L_Gem
Z_Im_L_Gem
Z_Shift_L_Cyt
Z_Im_L_Cyt

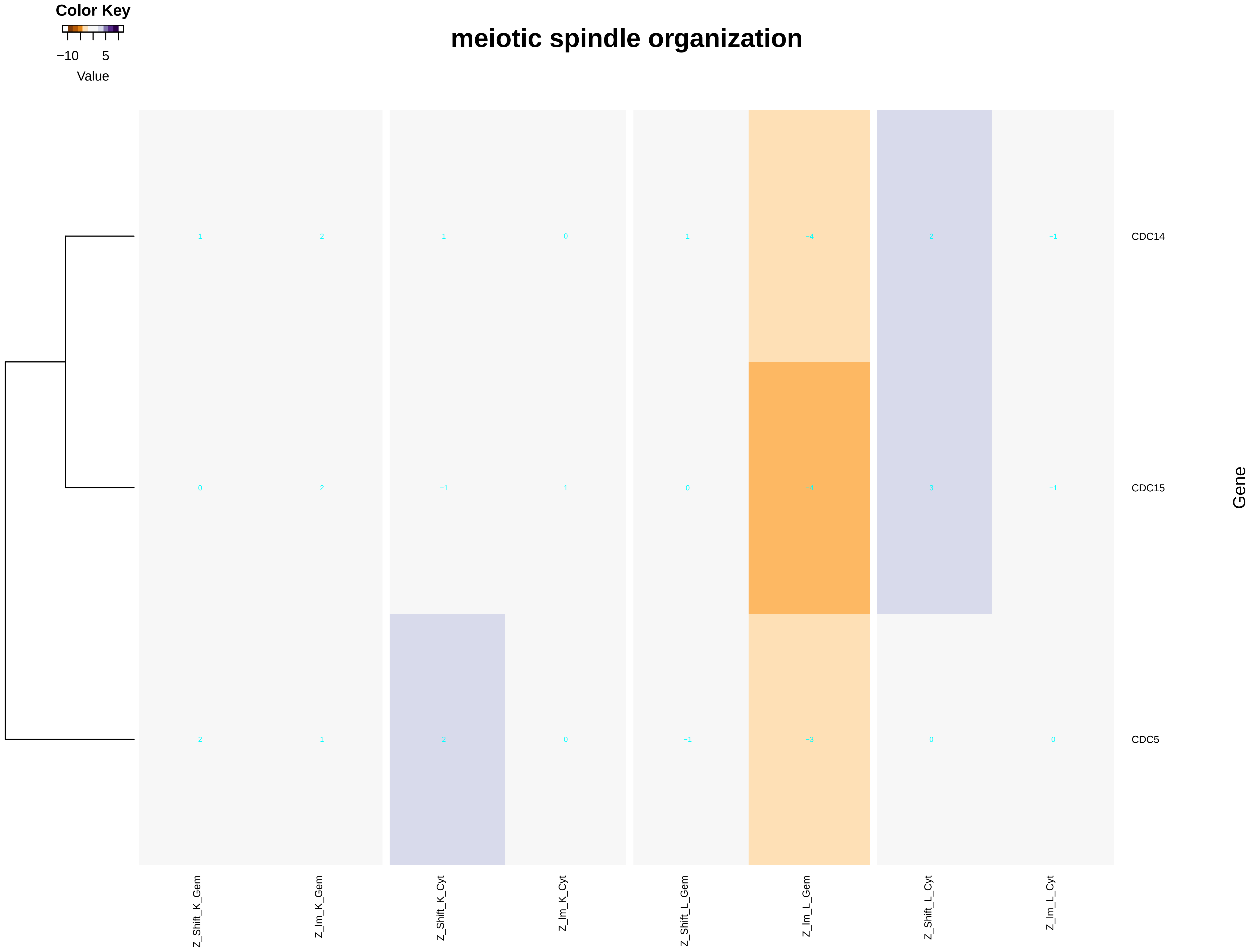
Color Key
−10
5
Value
meiotic spindle organization
1 2 1 0 1 −4 2 −1
0 2 −1 1 0 −4 3 −1
2 1 2 0 −1 −3 0 0
CDC14
CDC15
CDC5
Gene
Z_Shift_K_Gem
Z_Im_K_Gem
Z_Shift_K_Cyt
Z_Im_K_Cyt
Z_Shift_L_Gem
Z_Im_L_Gem
Z_Shift_L_Cyt
Z_Im_L_Cyt

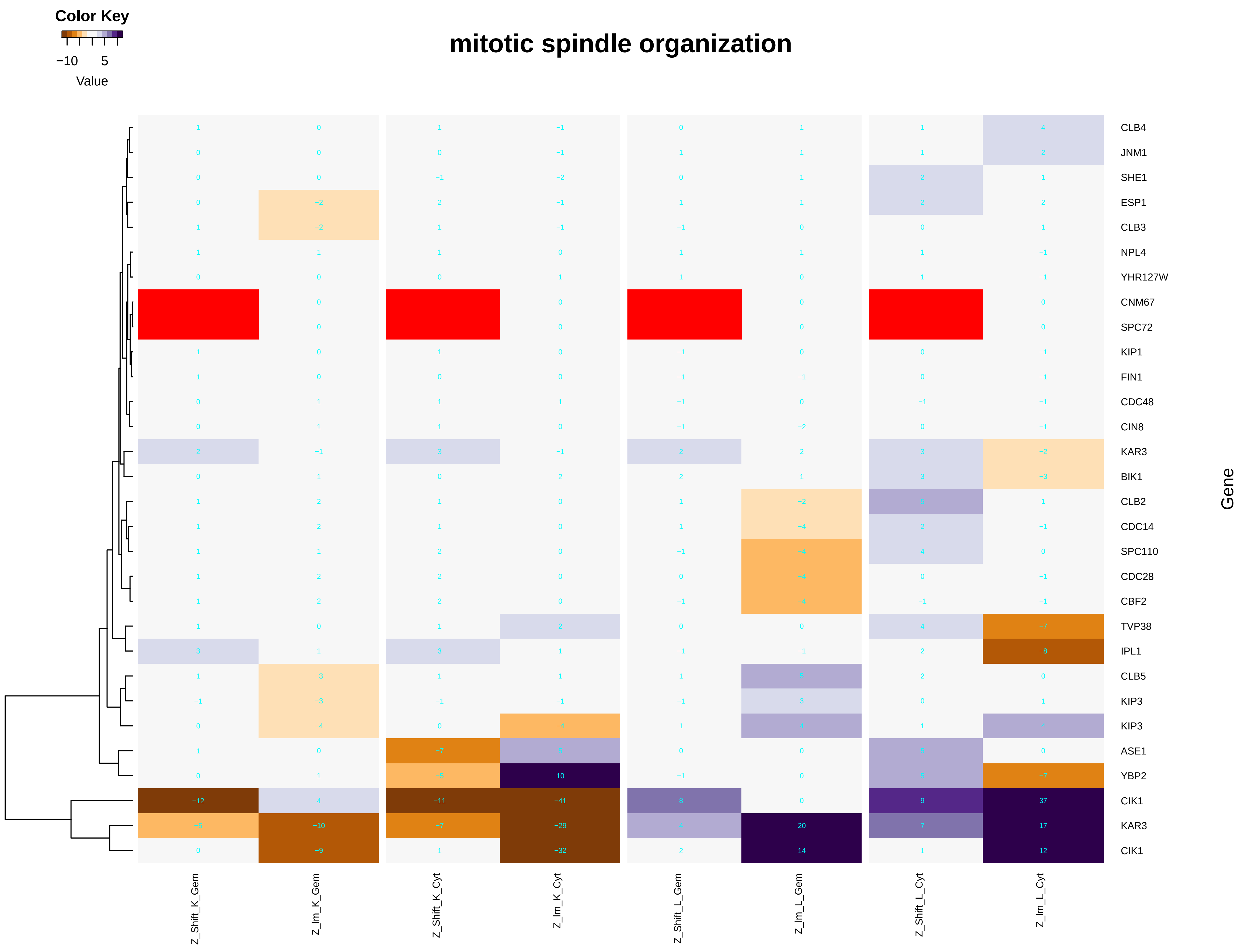
Color Key
−10
5
Value
mitotic spindle organization
CLB4
JNM1
SHE1
ESP1
CLB3
NPL4
YHR127W
CNM67
SPC72
KIP1
FIN1
CDC48
CIN8
KAR3
BIK1
CLB2
CDC14
SPC110
CDC28
CBF2
TVP38
IPL1
CLB5
KIP3
KIP3
ASE1
YBP2
CIK1
KAR3
CIK1
Gene
Z_Shift_K_Gem
Z_Im_K_Gem
Z_Shift_K_Cyt
Z_Im_K_Cyt
Z_Shift_L_Gem
Z_Im_L_Gem
Z_Shift_L_Cyt
Z_Im_L_Cyt

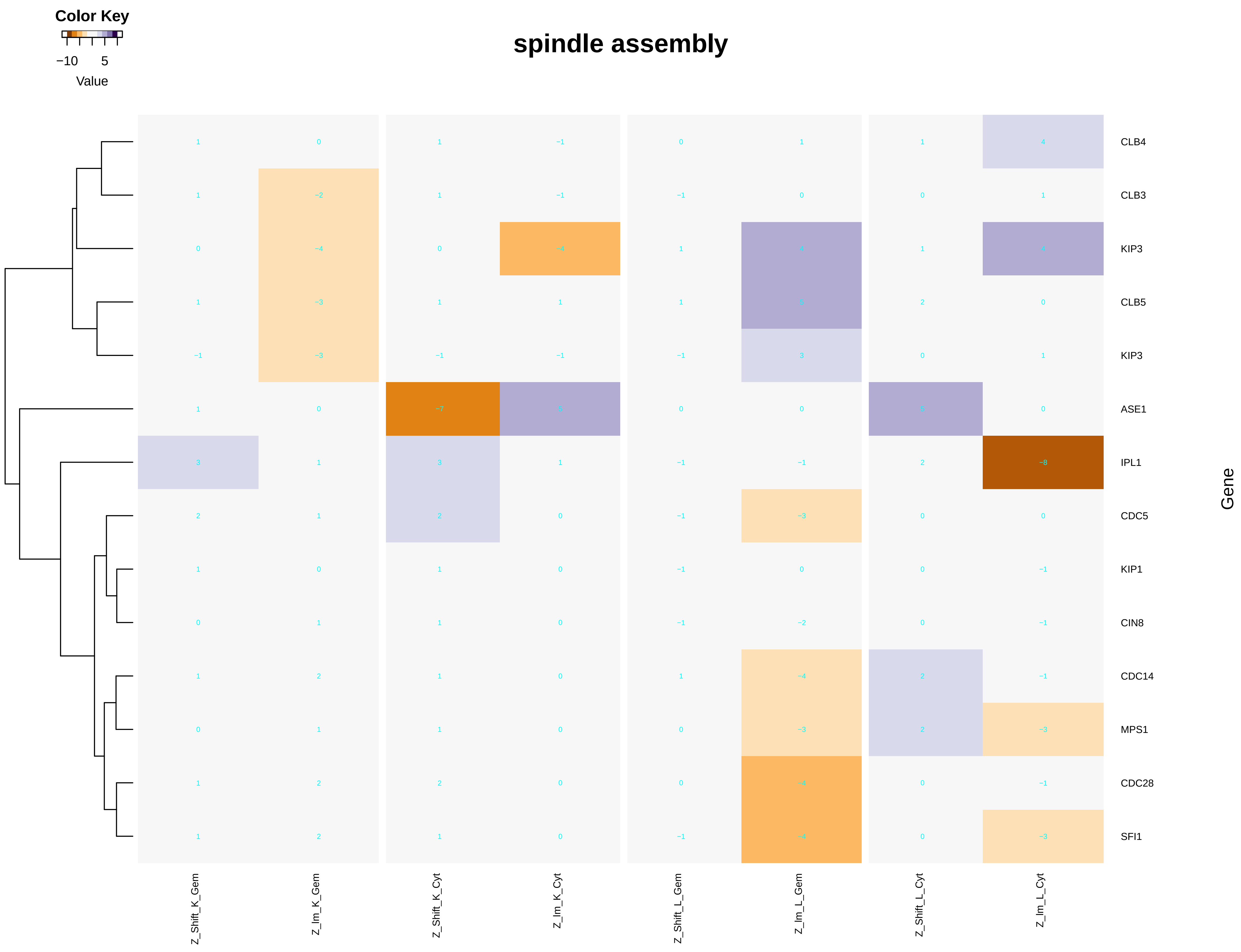
Color Key
−10 5
Value
spindle assembly
CLB4
CLB3
KIP3
CLB5
KIP3
ASE1
IPL1
CDC5
KIP1
CIN8
CDC14
MPS1
CDC28
SFI1
Gene
Z_Shift_K_Gem
Z_Im_K_Gem
Z_Shift_K_Cyt
Z_Im_K_Cyt
Z_Shift_L_Gem
Z_Im_L_Gem
Z_Shift_L_Cyt
Z_Im_L_Cyt

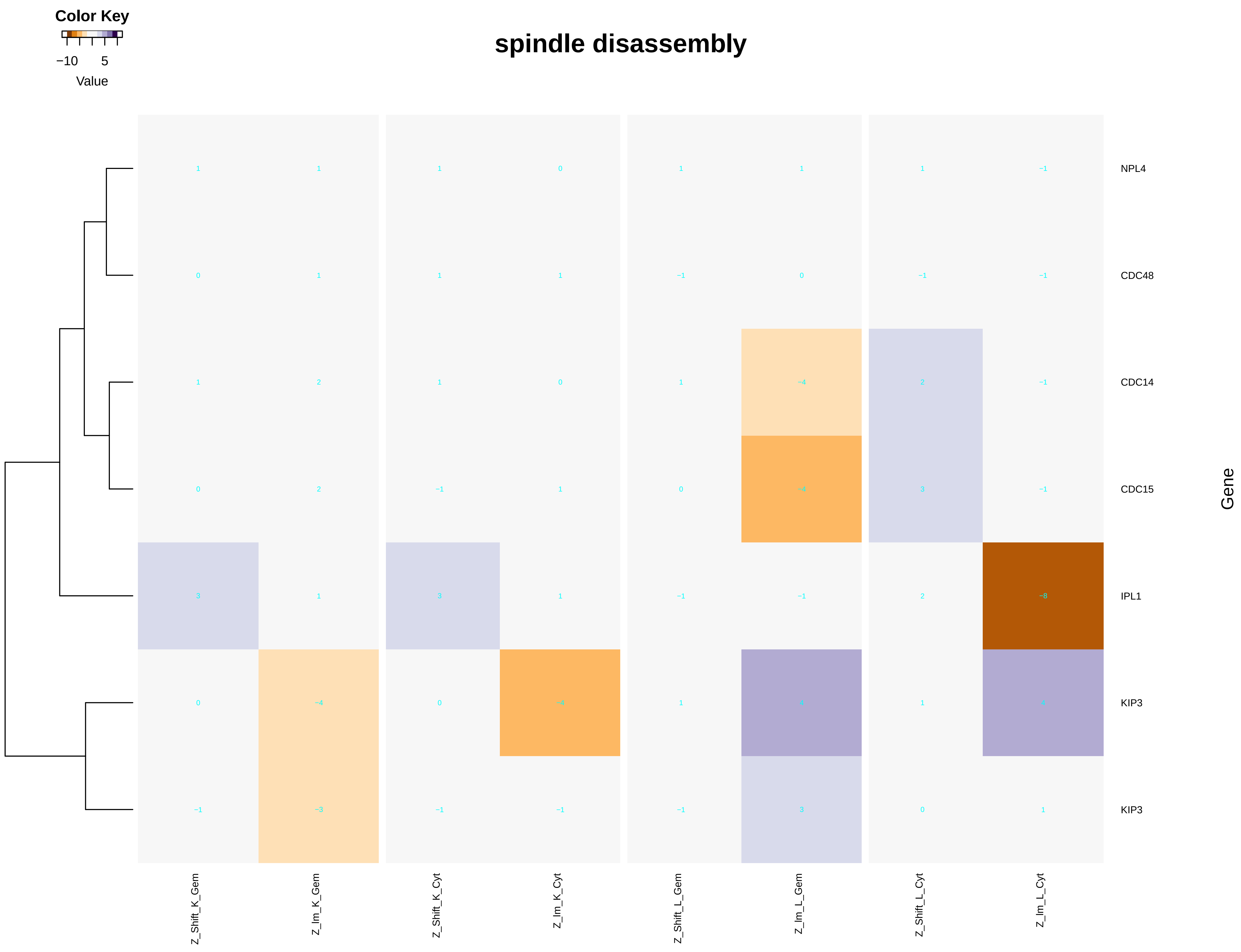

spindle disassembly
Color Key
−10
5
Value
NPL4
CDC48
CDC14
CDC15
IPL1
KIP3
KIP3
Gene
Z_Shift_K_Gem
Z_Im_K_Gem
Z_Shift_K_Cyt
Z_Im_K_Cyt
Z_Shift_L_Gem
Z_Im_L_Gem
Z_Shift_L_Cyt
Z_Im_L_Cyt

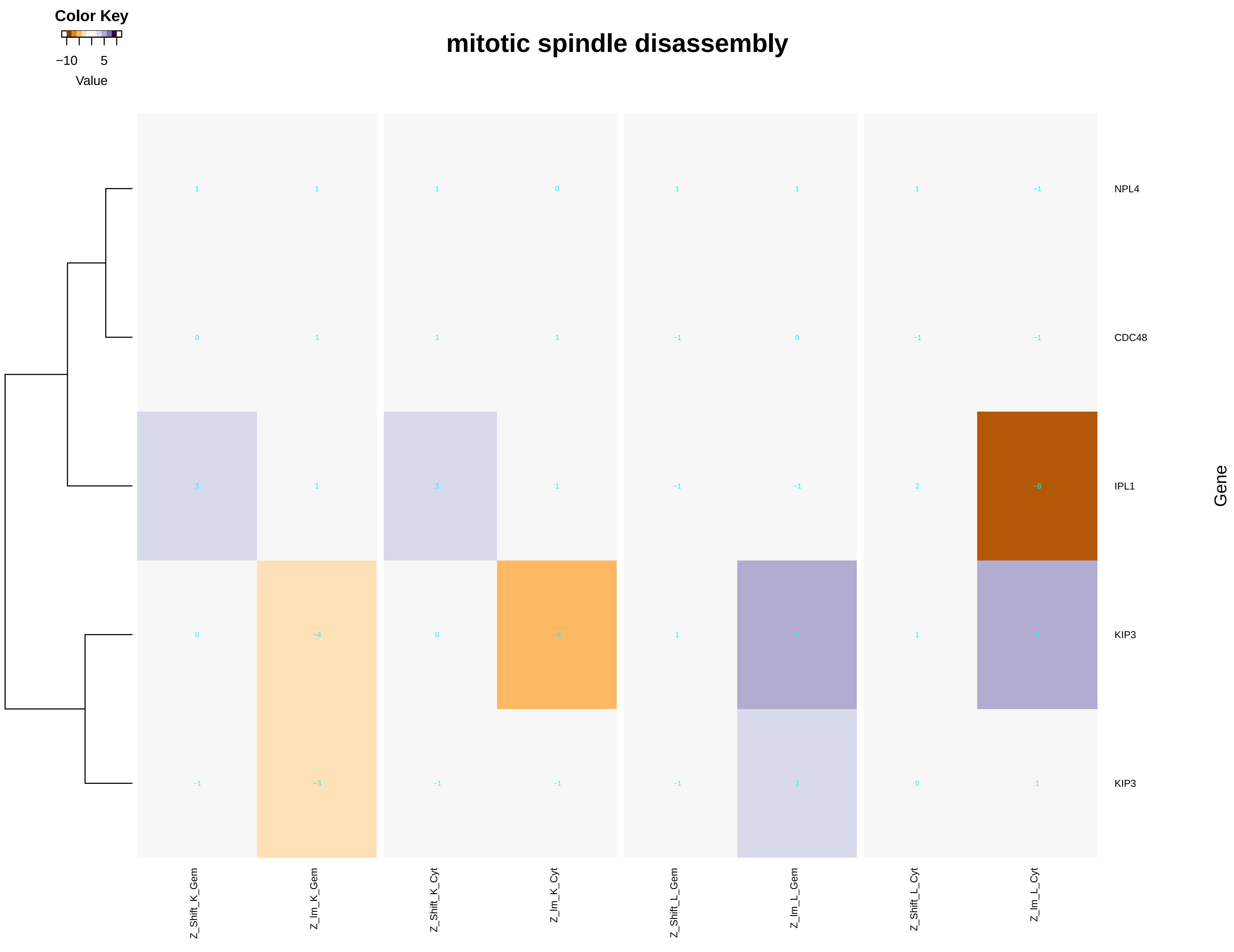
Color Key
−10 5
Value
mitotic spindle disassembly
NPL4
CDC48
IPL1
KIP3
KIP3
Gene
Z_Shift_K_Gem
Z_Im_K_Gem
Z_Shift_K_Cyt
Z_Im_K_Cyt
Z_Shift_L_Gem
Z_Im_L_Gem
Z_Shift_L_Cyt
Z_Im_L_Cyt

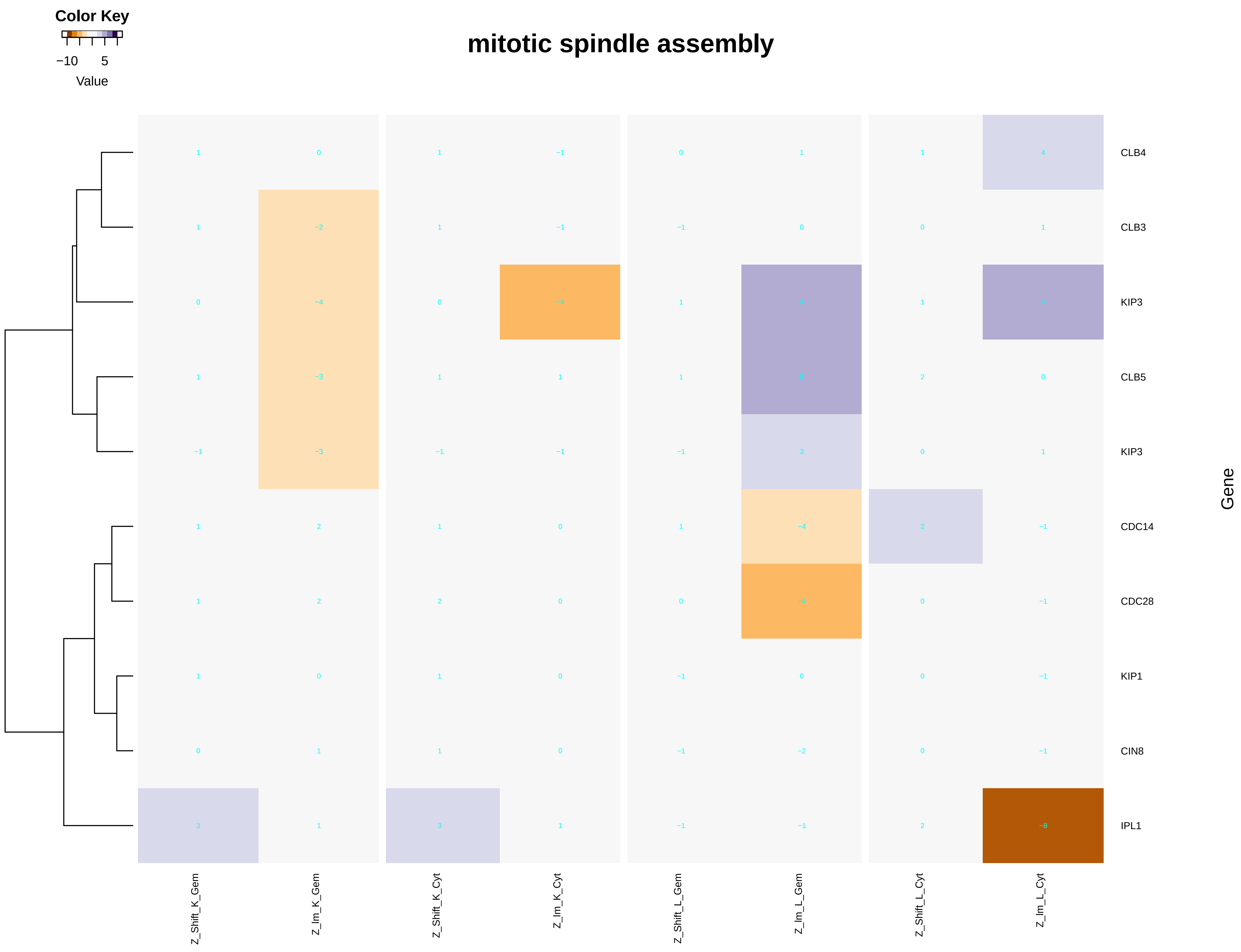
Color Key
−10
5
Value
mitotic spindle assembly
CLB4
CLB3
KIP3
CLB5
KIP3
CDC14
CDC28
KIP1
CIN8
IPL1
Gene
Z_Shift_K_Gem
Z_Im_K_Gem
Z_Shift_K_Cyt
Z_Im_K_Cyt
Z_Shift_L_Gem
Z_Im_L_Gem
Z_Shift_L_Cyt
Z_Im_L_Cyt
